# Chemical Composition and Insecticidal Activity of the Essential Oil of *Illicium pachyphyllum* Fruits against Two Grain Storage Insects

**DOI:** 10.3390/molecules171214870

**Published:** 2012-12-13

**Authors:** Peng Liu, Xin-Chao Liu, Hui-Wen Dong, Zhi-Long Liu, Shu-Shan Du, Zhi-Wei Deng

**Affiliations:** 1 Analytic and Testing Center, Beijing Normal University, Haidian District, Beijing 100875, China; 2 Department of Entomology, China Agricultural University, Haidian District, Beijing 100193, China; 3 Department of Biology, Taiyuan University, Taiyuan 030031, China; 4 College of Resources Science and Technology, Beijing Normal University, Haidian District, Beijing 100875, China

**Keywords:** *Illicium pachyphyllum*, *Sitophilus zeamais*, *Tribolium castaneum*, contact toxicity, fumigant, essential oil composition

## Abstract

The aim of this research was to determine chemical composition and insecticidal activity of the essential oil of *Illicium pachyphyllum* fruits against two grain storage insects, *Sitophilus zeamais* and *Tribolium castaneum*, and to isolate any insecticidal constituents from the essential oil. The essential oil of *I. pachyphyllum* fruits was obtained by hydrodistillation and analyzed by GC-MS. A total of 36 components of the essential oil were identified, with the principal compounds in the essential oil being *trans*-ρ-mentha-1(7),8-dien-2-ol (24.56%), d-limonene (9.79%), caryophyllene oxide (9.32%), and *cis*-carveol (5.26%) followed by β-caryophyllene (4.63%) and bornyl acetate. Based on bioactivity-guided fractionation, the three active constituents were isolated and identified as *trans*-ρ-mentha-1(7),8-dien-2-ol, d-limonene and caryophyllene oxide. The essential oil of *I. pachyphyllum* fruits exhibited contact toxicity against *S. zeamais* and *T. castaneum* adults, with LD_50_ values of 17.33 μg/adult and 28.94 μg/adult, respectively. *trans*-*p*-Mentha-1(7),8-dien-2-ol (LD_50_ = 8.66 μg/adult and 13.66 μg/adult, respectively) exhibited stronger acute toxicity against *S. zeamais* and *T. castaneum* adults than either caryophyllene oxide (LD_50_ = 34.09 μg/adult and 45.56 μg/adult) and d-limonene (LD_50_ = 29.86 μg/adult and 20.14 μg/adult). The essential oil of *I. pachyphyllum* possessed fumigant toxicity against *S. zeamais* and *T. castaneum* adults with LC_50_ values of 11.49 mg/L and 15.08 mg/L, respectively. *trans*-*p*-Mentha-1(7),8-dien-2-ol exhibited stronger fumigant toxicity against *S. zeamais* and *T. castaneum* adults, respectively, with LC_50_ values of 6.01 mg/L and 8.14 mg/L, than caryophyllene oxide (LC_50_ = 17.02 mg/L and 15.98 mg/L) and d-limonene (LC_50_ = 33.71 mg/L and 21.24 mg/L). The results indicate that the essential oil of *I. pachyphyllum* fruits and its constituent compounds have potential for development into natural insecticides or fumigants for the control of insects in stored grains.

## 1. Introduction

*Sitophilus* and *Tribolium* species are two of the major pests of stored grains and grain products in the tropics and subtropics [1]. Control of stored product insects relies heavily on the use of synthetic insecticides and fumigants, which has led to problems such as environmental disturbances, increasing costs of application, pest resistance to pesticides and consequent resurgence and lethal effects on non-target organisms, in addition to direct toxicity to users [2,3]. These problems have necessitated a search for alternative eco-friendly insect pest control methods [4]. Plant essential oils and their components have been shown to possess potential for development as new fumigants and they may have advantages over conventional fumigants in terms of low mammalian toxicity, rapid degradation and local availability [5]. Investigations in several countries confirm that some plant essential oils not only repel insects, but also possess contact and fumigant toxicity against stored product pests as well as exhibiting feeding inhibition or harmful effects on the reproductive system of insects [6,7]. Essential oils from many plants including medicinal herbs, spices and fruits have been evaluated with success for insecticidal activity against stored product insects/mites [8,9,10,11,12,13,14,15]. In some cases, they have been proven more effective than traditionally used organophosphorus pesticides. During our mass screening program for new agrochemicals from local wild plants and Chinese medicinal herbs, the essential oil from *Illicium pachyphyllum* A.C. Smith fruits has been found to possess insecticidal activity towards the maize weevil, *Sitophilus zeamais* (Motsch) and red flour beetle, *Tribolium castaneum* Herbst. 

*Illicium pachyphyllum* belongs to the family Illiciaceae and is an evergreen shrub, usually 2–3 m in height and mainly distributed in southern Guangxi Zhuang Autonomous Region and Yunnan Province [16]. Its fruits have 8–10 follicles which differentiates them from Chinese star anise (*I. verum*) with eight follicles. There are about 40 species in the Illiciaceae family, mostly found in East and Southeast Asia and 27 species (18 of them endemic) are found in China [16]. The essential oils derived from several Illiciaceae fruits exhibited insecticidal activity against insects, e.g., *I. verum* [17,18,19,20], *I. anisatum* [21], *I. difengpi* [22], *I. fargesii* [23], and *I. simonsii* [24]. However, a literature survey has shown that there is no report on the volatile constituents and insecticidal activity of *I. pachyphyllum*; thus we decided to investigate the chemical constituents and insecticidal activities of the essential oil of *I. pachyphyllum* against two grain storage insects for the first time and to isolate any active constituent compounds from the essential oil.

## 2. Results and Discussion

### 2.1. Essential oil Chemical Composition

**Figure 1:**
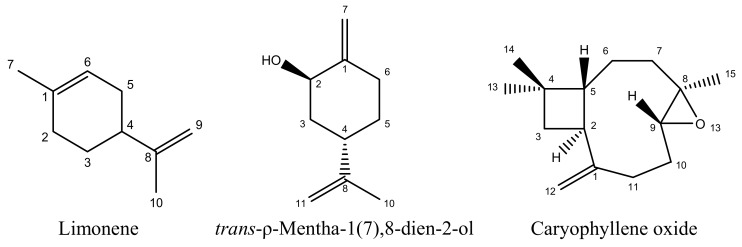
Constituent compounds isolated from the essential oil of *Illicium pachyphyllum* fruits.

The yield of yellow essential oil of *I. pachyphyllum* fruits was 1.21% (V/W) and the density of the concentrated essential oil was determined as 0.85 g/mL. A total of 36 components of the essential oil of *I. pachyphyllum* fruits were identified, accounting for 96.37% of the total oil (Table 1). The principal compounds in the essential oil were *trans*-ρ-mentha-1(7),8-dien-2-ol (24.56%), limonene (9.79%), caryophyllene oxide (9.32%) (Figure 1), and *cis*-carveol (5.26%), followed by β-caryophyllene (4.63%) and bornyl acetate. Monoterpenoids represented 24 of the 36 compounds, corresponding to 73.86% of the whole oil, while 11 of the 36 constituents were sesquiterpenoids (22.18% of the crude essential oil). 

### 2.2. Insecticidal Activities

The essential oil of *I. pachyphyllum* fruits exhibited contact toxicity against *S. zeamais* and *T. castaneum* adults with LD_50_ values of 17.33 μg/adult and 28.94 μg/adult, respectively (Table 2). When compared with the positive control pyrethrum extract (25% pyrethrine I and pyrethrine II), the essential oil of demonstrated only four and 80 times less acute toxicity against the two species of grain storage insects because the pyrethrum extract has acute toxicity to *S. zeamais* and *T. castaneum* with LD_50_ values of 4.29 μg/adult and 0.36 μg/adult, respectively [9].

Howover, compared with the other essential oils reported in the previous studies, the essential oil of *I. pachyphyllum* fruits exhibited stronger acute toxicity against the maize weevils, e.g., essential oils of *Artemisia capillaris* (LD_50_ = 105.95 μg/adult) and *A. mongolica* (LD_50_ = 87.92 μg/adult) [25], *A. vestita* (LD_50_ = 50.62 μg/adult) [8], *I. fragesii* fruits (LD_50_ = 28.95 μg/adult) [23], *I. simonsii* (LD_50_ = 112.74 μg/adult) [24]. The essential oil shows less acute toxic against the maize weevils than *I. difengpi* essential oil (LD_50_ = 13.83 μg/adult) [22]. 

*trans*-ρ-Mentha-1(7),8-dien-2-ol exhibited stronger acute toxicity against *S. zeamais* and *T. castaneum* adults (LD_50_ = 8.66 μg/adult and 13.66 μg/adult, respectively) than caryophyllene oxide (LD_50_ = 34.09 μg/adult and 45.56 μg/adult) and limonene (LD_50_ = 29.86 μg/adult and 20.14 μg/adult) (Table 2). *trans*-ρ-Mentha-1(7),8-dien-2-ol possessed almost four times more toxicity than caryophyllene oxide and limonene against *S. zeamais* adults and also was two times more toxic to the maize weevils than the crude essential oil (Table 2). It is suggested that the contact toxicity of the essential oil of *I. pachyphyllum* fruits may be attributed to *trans*-ρ-mentha-1(7),8-dien-2-ol. However, compared with pyrethrum extract (positive control), *trans*-ρ-mentha-1(7),8-dien-2-ol showed two times and 38 times less toxicity against *S. zeamais* and *T. castaneum* adults, respectively. 

The essential oil of *I. pachyphyllum* fruits possessed fumigant toxicity against *S. zeamais* and *T. castaneum* adults with LC_50_ values of 11.49 mg/L air and 15.08 mg/L air, respectively (Table 3). The commercial grain fumigant, methyl bromide (MeBr) was reported to have fumigant activity against *S. zeamais* and *T. castaneum* adults with LC_50_ values of 0.67 mg/L and 1.75 mg/L air, respectively [1], thus the essential oil of *I. pachyphyllum* fruits was 17 and 22 times less toxic to *S. zeamais* and *T. castaneum*, respectively. 

However, considering the commercial fumigants are synthetic insecticides and the most effective fumigants (e.g., phosphine and MeBr) are also highly toxic to humans and other non-target organisms, fumigant activity of the essential oil of *I. pachyphyllum* fruits is quite promising. Moreover, compared with the other essential oils in the previous studies, the essential oil of *I. pachyphyllum* exhibited stronger or same level of fumigant toxicity against the maize weevils, e.g., essential oils of *A. lavandulaefolia* (LC_50_ = 11.2 mg/L) and *A. sieversiana* (LC_50_ = 15.0 mg/L) [26], *A. vestita* (LC_50_ = 13.42 mg/L) [8], *I. simonsii* (LC_50_ = 14.95 mg/L) [24], and *Kadsura heteroclite* (LC_50_ = 14.01 mg/L) [27]. Compared with the other essential oils reported in the literature, the essential oil of *I. pachyphyllum* possessed stronger fumigant toxicity against *T. castaneum* adults, e.g., essential oils of *Citrus reticulata* (LC_50_ = 19.47 μL/L) and *Schinus terebenthifolius* (LC_50_ = 20.50 μL/L) [28], *Perovskia abrotanoides* (LC_50_ = 11.39 μL/L) [29], and *Drimys winteri* (LC_50_ = 9.0–10.5 μL/L) [30], but lesser toxicity than the essential oil of *Laurelia sempervirens* (LC_50_ = 1.6–1.7 μL/L) [30].

Among the three constituent compounds, *trans*-ρ-mentha-1(7),8-dien-2-ol exhibited stronger fumigant toxicity against *S. zeamais* and *T. castaneum* adults with LC_50_ values of 6.01 mg/L air and 8.14 mg/L air, respectively than caryophyllene oxide (LC_50_ = 17.02 mg/L and 15.98 mg/L, respectively) and limonene (LC_50_ = 33.71 mg/L and 21.24 mg/L, respectively) (Table 3). *trans*-ρ-Mentha-1(7),8-dien-2-ol was almost six times and three times more toxic than limonene to *S. zeamais* and *T. castaneum* adults, respectively, and also two times more toxic to the two species of stored product insects than the crude essential oil. However, compared with MeBr (positive control), *trans*-ρ-mentha-1(7),8-dien-2-ol showed only nine times and five times less toxicity against *S. zeamais* and *T. castaneum* adults, respectively. 

There is no reports so far on the insecticidal activity of *trans*-ρ-mentha-1(7),8-dien-2-ol. However, in previous reports, caryophyllene oxide was found to exhibit insecticidal and antifeedant activity [31]. Caryophyllene oxide acts as a nerve poison to pests via sodium channel modulators [32]. Limonene is a phagostimulant disruptor which inhibits and breaks the developed pest resistance against pesticides because it destroys the wax that coats the inside of the insects respiratory system [32]. Limonene has been commercialized for use as flea dips and shampoos for pets as well as sprays and aerosols [33]. It has been demonstrated to possess insecticidal activity against several stored-product insects such as the cowpea weevil (*C. maculates*), lesser grain borer (*R. dominica*), flat grain beetle (*C. pusillus*), rice weevil (*S. oryzae*), maize weevil (*S. zeamais*) and red flour beetle (*T. castaneum*), as well as against the stored food mite *Tyrophagus putrescentiae* [34,35,36,37,38]. Limonene was demonstrated to be a potent inhibitor of acetylcholinesterase (AChE) activity in larvae of several stored product insects [34].

The above findings suggest that that insecticidal activity especially fumigant activity of the essential oil of *I. pachyphyllum* and its three constituent compounds against the two grain storage insects is quite promising. The essential oil of *I. pachyphyllum* and its three constituent compounds, especially *trans*-ρ-mentha-1(7),8-dien-2-ol, show potential to be developed as possible natural fumigants/insecticides for the control of grain storage insects. However, to develop a practical application for the essential oil and the isolated constituents as novel fumigants/insecticides, further research into the safety of the essential oil/compounds to humans is needed. Additional studies on the development of formulations are also necessary to improve the efficacy and stability and to reduce cost.

## 3. Experimental

### 3.1. Plant Material and Essential Oil Extraction

The fresh fruits of *I. pachyphyllum* (6 kg) were harvested at November 2011 from Wenshan County (23.37° N latitude and 104.25° E longitude, Yunnan Province, China). The plant was identified by Dr. Liu, QR (College of Life Sciences, Beijing Normal University, China) and a voucher specimen (CMH-Houpibajiao-Yuannan-2011-11) was deposited in the museum of Department of Entomology, China Agricultural University. The sample was air-dried and ground to a powder using a grinding mill (Retsch Muhle, Haan, Germany). The powder was subjected to hydrodistillation using a modified Clevenger-type apparatus for 6 h and extracted with *n*-hexane. Anhydrous sodium sulphate was used to remove water after extraction. The essential oil was stored in airtight containers in a refrigerator at 4 °C for subsequent experiments.

### 3.2. Insects

The maize weevils (*S. zeamais*) and red flour beetles (*T. castaneum*) were obtained from laboratory cultures maintained in the dark in incubators at 29–30 °C and 70%–80% r.h. The red flour beetles were reared on wheat flour mixed with yeast (10:1, w/w) while maize weevils were reared on whole wheat at 12%–13% moisture content. Unsexed adult weevils/beetles used in all the experiments were about two weeks old.

### 3.3. Gas Chromatography-Mass Spectrometry

Components of the essential oil of *I. pachyphyllum* fruits were separated and identified by gas chromatography-flame ionization detection (GC-FID) and gas chromatography-mass spectrometry (GC-MS) using an Agilent 6890N gas chromatograph connected to an Agilent 5973N mass selective detector. The same column and analysis conditions were used for both GC-FID and GC-MS. They were equipped with capillary column with HP-5MS (30 m × 0.25 mm × 0.25 μm). The GC settings were as follows: the initial oven temperature was held at 60 °C for 1 min and ramped at 10 °C·min^−1^ to 180 °C where it was held for 1 min, and then ramped at 20 °C·min^−1^ to 280 °C and held there for 15 min. The injector temperature was maintained at 270 °C. The samples (1 μL, dilute to 1% with acetone) were injected, with a split ratio of 1:10. The carrier gas was helium at flow rate of 1.0 mL·min^−1^. Spectra were scanned from 20 to 550 *m/z* at 2 scans·s^−1^. Most constituents were identified by gas chromatography by comparison of their retention indices with those of the literature or with those of authentic compounds available in our laboratories. The retention indices were determined in relation to a homologous series of *n*-alkanes (C_8_–C_24_) under the same operating conditions. Further identification was made by comparison of their mass spectra with those stored in NIST 05 (Standard Reference Data, Gaithersburg, MD, USA) and Wiley 275 libraries (Wiley, New York, NY, USA) or with mass spectra from literature [39]. Component relative percentages were calculated based on GC peak areas without using correction factors.

### 3.4. Purification and Characterization of Three Constituent Compounds 

The crude essential oil of *I. pachyphyllum* fruits (25 mL) was chromatographed on a silica gel (Merck 9385) column (85 mm i.d., 850 mm length, 1,000 g) by gradient elution with a mixture of solvents (*n*-hexane, *n*-hexane-ethyl acetate). Fractions of 500 mL were collected and concentrated at 40 °C, and similar fractions according to TLC profiles were combined to yield 18 fractions. Fractions (3–5, 8, 12) that possessed contact toxicity, with similar TLC profiles, were pooled and further purified by preparative silica gel column chromatography (PTLC) with petroleum ether-acetone (50:1, v/v) until to obtain the pure compound for determining structure as limonene (0.5 g), *trans-*ρ-mentha-1(7),8-dien-2-ol (0.3 g) and caryophyllene oxide (0.7 g). The structure of the compounds was elucidated based on nuclear magnetic resonance. ^1^H and ^13^C-NMR spectra were recorded on Bruker AMX500 [500 MHz (^1^H)] instruments using CDCl_3_ or DMSO-d_6_ as the solvent with TMS as internal standard. 

### 3.5. Isolated Constituent Compounds

*Limonene* (Figure 1), yellow oil, C_10_H_12_O. ^1^H-NMR (DMSO-*d_6_*, 500 MHz) δ: 5.33–5.49 (1H, m, H-6), 4.73 (2H, s, H-9), 2.03–2.18 (3H, m, H-2, H-4), 1.88–2.02 (2H, m, H-5), 1.79–1.86 (1H, m, H-3a), 1.76 (3H, s, H-10), 1.68 (3H, s, H-7), 1.50 (1H, dd, *J* = 12 and 6 Hz, H-3b). ^13^C-NMR (DMSO-*d_6_*, 125 MHz) δ: 149.8 (C-8), 133.5 (C-1), 120.9 (C-6), 109.2 (C-9), 40.9 (C-4), 30.6 (C-2), 30.5 (C-5), 27.8 (C-3), 23.7 (C-7), 21.2 (C-10). The data matched a previous report [40]. 

*trans-ρ-Mentha-1(7),8-dien-2-ol* (Figure 1), A colorless oil, C_10_H_16_O. ^1^H-NMR (CDCl_3_, 500 MHz) δ: 4.85 (1H, s, H-7), 4.77 (1H, s, H-7), 4.72 (2H, br. s., H-9), 4.37 (1H, br. s., H-2), 2.50–2.59 (1H, m, H-4), 2.45–2.50 (1H, m, H-6), 2.21 (1H, dt, *J* = 14 and 3 Hz, H-6), 1.97–2.02 (1H, m, H-3), 1.86–1.89 (1H, m, OH), 1.82–1.86 (1H, m, H-5), 1.73 (3H, s, H-10), 1.48–1.56 (1H, m, H-3), 1.29 (1H, m, H-5). ^13^C-NMR (CDCl_3_, 125 MHz) δ: 149.8 (C-1), 149.4 (C-8), 109.9 (C-7), 108.9 (C-9), 72.4 (C-2), 39.0 (C-3), 38.1 (C-4), 32.6 (C-5), 29.9 (C-6), 21.0 (C-10). The data matched a previous report [41].

*Caryophyllene oxide* (Figure 1), colorless oil, C_15_H_24_O. ^1^H-NMR (CDCl_3_, 500 MHz) δ: 5.00 (1H, *s*, H-12), 4.88 (1H, *s*, H-12), 2.90 (1H, *dd*, *J* = 4.1 and 10.7 Hz, H-9), 2.64 (1H, *d*, *J* = 9.1 Hz, H-2), 2.33–2.37 (1H, *m*, H-11), 2.28 (1H, *dd*, *J* = 3.6 and 8.4 Hz, H-10), 2.09–2.15 (2H, *m*, H-7, H-11), 1.72–1.74 (1H, *m*, H-5), 1.69 (1H, *br. s.*, H-3), 1.65–1.67 (1H, *m*, H-6), 1.62 (1H, *br. s*, H-3), 1.44 (1H, *d*, *J* = 2.8 Hz, H-6), 1.36–1.39 (1H, *m*, H-10), 1.23 (3H, *s*, H-15), 1.03 (3H, *s*, H-13), 1.01 (3H, *s*, H-14), 0.99 (1H, *br. s.*, H-7). ^13^C-NMR (CDCl_3_, 125 MHz) δ: 151.8 (C-1), 112.8 (C-12), 63.8 (C-9), 59.9 (C-8), 50.7 (C-5), 48.9 (C-2), 39.7 (C-3), 39.1 (C-7), 34.0 (C-4), 30.2 (C-10), 29.9 (C-14), 29.8 (C-11), 27.2 (C-6), 21.6 (C-13), 17.0 (C-15). The data matched a previous report [42].

### 3.6. Fumigant Toxicity

The fumigant activity of the essential oil and the pure compounds against *S. zeamais* and *T. castaneum* adults was tested as described by Liu and Ho [1]. Range-finding studies were run to determine the appropriate testing concentrations. A serial dilution of the essential oil/compound (4–40.0 mg/L for pure compounds, 5–50 mg/L for oil, five concentrations) was prepared in *n*-hexane. A Whatman filter paper (diameter 2.0 cm) were each impregnated with 10 μL dilution, and then placed on the underside of the screw cap of a glass vial (diameter 2.5 cm, height 5.5 cm, volume 25 mL). The solvent was allowed to evaporate for 15 s before the cap was placed tightly on the glass vial, each of which contained 10 insects inside to form a sealed chamber. Fluon (ICI America Inc.) was used inside the glass vial to prevent insects from contacting the treated filter paper. Preliminary experiments demonstrated that 15 s was sufficient for the evaporation of solvents. *n*-Hexane was used as a control. Five replicates were carried out for all treatments and controls, and they were incubated for 24 h. The insects were then transferred to clean vials with some culture media and returned to the incubator and observed daily for determination of end-point mortality, which was reached after one week. The experiments were repeated in three times. The observed data were corrected for control mortality using Abbott’s formula and LC_50_ values were calculated by using Probit analysis [43]. 

### 3.7. Contact Toxicity

The contact toxicity of the essential oil/pure compounds against *S. zeamais* and *T. castaneum* adults was measured as described by Liu and Ho [1]. Range-finding studies were run to determine the appropriate testing concentrations. A serial dilution of the essential oil and isolated compounds [4.0%–8.0% (approximately equal to 20–40 μg/adult) for pure compounds, 1.50%–5.20% (approximately equal to 7.5–26 μg/adult) for oil, five concentrations] was prepared in *n*-hexane. Aliquots of 0.5 μL of the dilutions were applied topically to the dorsal thorax of the insects, using a Burkard Arnold microapplicator. Controls were determined using *n*-hexane. Five replicates were carried out for all treatments and controls. Both treated and control insects were then transferred to glass vials (10 insects/vial) with culture media and kept in incubators. Mortality of insects was observed daily until end-point mortality was reached one week after treatment. The experiments were repeated in three times. The observed data were corrected for control mortality using Abbott’s formula and the results from all replicates were subjected to probit analysis using the PriProbit Program V1.6.3 to determine LC_50_ values [43]. Pyrethrum extract (25% pyrethrine I and pyrethrine II) was purchased from Fluka Chemie.

## 4. Conclusions

The study indicates that the essential oil of *I. pachyphyllum* fruits and its constituent compounds, *trans-*ρ-mentha-1(7),8-dien-2-ol, d-limonene, and caryophyllene oxide have potential for development into natural insecticides/fumigants for control of insects in stored grains.

## Figures and Tables

**Table 1:** Chemical constituents of the essential oil derived from *Illicium pachyphyllum* fruits.

	RI *	Compound	Composition %
1	933	α-pinene	2.49
2	954	camphene	1.07
3	961	benzaldehyde	0.33
4	974	β-pinene	0.29
5	991	β-myrcene	0.18
6	1026	ρ-cymene	0.29
7	1029	limonene	9.79
8	1031	1,8-cineole	3.51
9	1088	*trans*-linalool oxide	1.53
10	1090	fenchol	0.73
11	1094	linalool	0.45
12	1117	*trans*-(−)-pinocarveol	3.78
13	1140	camphor	2.01
14	1146	isoborneol	0.31
15	1162	borneol	0.78
16	1167	4-terpineol	1.13
17	1179	*trans*-ρ-mentha-1(7),8-dien-2-ol	24.56
18	1182	α-terpineol	1.13
19	1185	*p*-cymen-8-ol	0.86
20	1189	myrtenol	1.66
21	1192	verbenone	1.03
22	1204	*cis*-ρ-mentha-1(7),8-dien-2-ol	3.42
23	1220	carvone	3.26
24	1229	*cis*-carveol	5.59
25	1238	bornyl acetate	4.01
26	1285	α-copaene	1.05
27	1375	β-elemene	1.43
28	1394	β-caryophyllene	4.63
29	1420	γ-elemene	1.18
30	1433	α-caryophyllene	1.25
31	1454	γ-muurolene	0.72
32	1477	β-ionone	1.54
33	1484	eremophilene	0.66
34	1502	caryophyllene oxide	9.23
35	1583	γ-eudesmol	0.17
36	1648	β-eudesmol	0.32
		Total identified	96.37
		Monoterpenoids	73.86
		Sesquiterpenoids	22.18
		Others	0.33

* RI: retention index as determined on a HP-5MS column using the homologous series of *n*-hydrocarbons.

**Table 2:** Contact toxicity of the essential oil of *Illicium pachyphyllum* fruits and its constituents against *Sitophilus zeamais* and *Tribolium castaneum* adults.

Insects	Treatment	LD_50_ (μg/adult)	95% FL *	Slope ± SE	Chi square (χ^2^)
*Sitophilus zeamais*	*I. pachyphyllum*	17.33	15.97–18.78	5.37 ± 0.51	9.84
	Caryophyllene oxide	34.09	28.98–38.61	1.88 ± 0.22	14.76
	Limonene	29.86	27.28–30.10	7.38 ± 0.84	8.64
	*trans*-ρ-Mentha-1(7),8-dien-2-ol	8.46	7.58–9.38	3.65 ± 0.38	10.64
	Pyrethrum extract	4.29	3.86–4.72	-	
*Tribolium castaneum*	*I. pachyphyllum*	28.94	25.67–32.74	3.05 ± 0.35	9.24
	Caryophyllene oxide	45.56	38.24–52.29	2.13 ± 0.23	8.12
	Limonene	20.14	18.45–21.89	1.59 ± 0.22	16.02
	*trans*-ρ-Mentha-1(7),8-dien-2-ol	13.66	12.36–15.27	3.99 ± 0.41	8.40
	Pyrethrum extract	0.36	0.32–0.41	6.87 ± 0.77	-

* Fiducial limits.

**Table 3:** Fumigant toxicity of the essential oil of *Illicium pachyphyllum* fruits and its constituents against *Sitophilus zeamais* and *Tribolium castaneum* adults.

Insects	Treatment	LC_50_ (mg/L)	95% FL *	Slope ± SE	Chi square (χ^2^)
*Sitophilus zeamais*	*I. pachyphyllum*	11.49	10.16–12.79	3.58 ± 0.36	9.84
	Caryophyllene oxide	17.02	15.52–18.83	2.16 ± 0.26	20.16
	Limonene	33.71	30.70–36.28	3.61 ± 0.40	14.88
	*trans*-ρ-Mentha-1(7),8-dien-2-ol	6.01	5.33–6.68	4.10 ± 0.42	10.36
	MeBr **	0.67	-	-	
*Tribolium castaneum*	*I. pachyphyllum*	15.08	13.59–16.67	3.76 ± 0.38	9.24
	Caryophyllene oxide	15.98	13.79–17.77	5.32 ± 0.57	10.08
	Limonene	21.24	19.03–22.14	6.21 ± 0.59	15.84
	*trans*-ρ-Mentha-1(7),8-dien-2-ol	8.14	7.03–9.31	3.20 ± 0.31	7.84
	MeBr **	1.75	-	-	-

* Fiducial limits; ** data from Liu and Ho [1].
